# Dapaglifozin use in pediatric IgA nephropathy: a single-center real-life experience

**DOI:** 10.1093/ckj/sfaf395

**Published:** 2025-12-13

**Authors:** Luigi Annicchiarico Petruzzelli, Gabriele Malgieri, Martina Carucci, Oriana De Marco

**Affiliations:** Department of Pediatric Specialties, Pediatric Nephrology, Dialysis and Renal Transplantation Santobono Pausilipon Children’s Hospital, Naples, Italy; Department of Pediatric Specialties, Pediatric Nephrology, Dialysis and Renal Transplantation Santobono Pausilipon Children’s Hospital, Naples, Italy; Department of Pediatric Specialties, Pediatric Nephrology, Dialysis and Renal Transplantation Santobono Pausilipon Children’s Hospital, Naples, Italy; Department of Pediatric Specialties, Pediatric Nephrology, Dialysis and Renal Transplantation Santobono Pausilipon Children’s Hospital, Naples, Italy; Department of Mental and Physical Health and Preventive Medicine, University of Campania “Luigi Vanvitelli”, Naples, Italy

To the Editor,

Immunoglobulin A nephropathy (IgAN) represents the leading cause of primary glomerulonephritis worldwide and exhibits a heterogeneous presentation, including microscopic or macroscopic hematuria, variable degrees of proteinuria and the potential for long-term renal decline [1]. While renin–angiotensin system (RAS) inhibition forms the basis of current supportive management, a substantial subset of pediatric patients continues to experience persistent proteinuria despite maximized therapy [2]. In recent years, sodium–glucose cotransporter-2 inhibitors (SGLT2i) have emerged as promising renoprotective agents in adults with chronic kidney disease, including IgAN [3, 4]. Nevertheless, pediatric data on SGLT2i are virtually absent, and their safety and effectiveness in children remain undefined [5].

We conducted a prospective single-center study evaluating the use of dapagliflozin in nine pediatric patients with biopsy-proven IgAN. The cohort included seven males and two females (median age 14 years). All patients were on angiotensin-converting enzyme inhibitor/angiotensin-receptor blocker therapy; eight had M1E0S0T0C0 lesions and one had C2 lesions. Dapagliflozin was administered at 5 mg/day, with follow-up assessments performed at 1 month (T1), 3 months (T2) and 6 months (T3) (Supplementary data, Table S1).

Renal function showed a transient hemodynamic change during dapagliflozin therapy. Serum creatinine increased modestly at 1–3 months but returned to near baseline values by 6 months (0.65 vs 0.60 mg/dL; *P* = .25). Similarly, estimated glomerular filtration rate showed an initial decline followed by recovery to above-baseline levels (171 vs 158 mL/min/1.73 m²; *P* = .20). These mild fluctuations reflect the well-known hemodynamic adjustments induced by SGLT2 inhibition in adults and did not translate into long-term renal impairment (Table 1). Proteinuria demonstrated a marked and consistent reduction. The median urine protein/creatinine ratio decreased from 0.46 at baseline to 0.12 at 6 months (*P* = .0039), while 24-h proteinuria dropped from 570 mg/day to 157 mg/day (*P* = .0039). These antiproteinuric effects appeared early and were sustained throughout follow-up (Table 1).

**Table 1:** Renal outcomes during 6 months of dapagliflozin treatment.

Variable	T0	T1	T2	T3	*P* (Friedman)	*P* (T0 vs T3)
Serum creatinine (mg/dL)	0.60 (0.54–0.65)	0.67 (0.63–0.75)	0.72 (0.62–0.75)	0.65 (0.58–0.67)	.0013	.25 (ns)
eGFR (mL/min/1.73 m²)	158 (146–180)	145 (135–160)	150 (147–159)	171 (159–176)	.0011	.20 (ns)
uP/uC ratio	0.46 (0.41–0.81)	0.18 (0.10–0.22)	0.15 (0.10–0.24)	0.12 (0.10–0.15)	.00077	.0039
24-h proteinuria (mg/day)	570 (554–596)	208 (155–253)	158 (141–172)	157 (105–165)	.00023	.0039

Values expressed as median and interquartile range.

*P*-values refer to Friedman’s test (global time effect) and Wilcoxon signed-rank test comparing baseline vs 12 months; ‘ns’ indicates non-significant.

Timepoints: T0 = baseline; T1 = 1 month; T2 = 3 months; T3 = 6 months.

eGFR, estimated glomerular filtration rate; uP/uC, urine protein/creatinine ratio.

Dapagliflozin was well tolerated. Glucosuria occurred, as expected, yet no patient experienced urinary tract infections, dehydration or clinically relevant metabolic disturbances. Ketonuria was mild and transient. Blood pressure, body mass index and glucose levels remained stable. Urine output increased in all patients during dapagliflozin therapy, with a mean rise of approximately 856 mL from baseline to Month 6 (from 1339 mL to 2194 mL), while body weight remained stable throughout follow-up. No patient discontinued therapy, supporting a favorable safety profile (Supplementary data, Table S2).

In conclusion, our findings provide preliminary evidence that dapagliflozin may represent a safe and potentially effective adjunctive therapy for pediatric IgAN, particularly in children with persistent proteinuria despite optimized RAS inhibition. Evidence on the use of SGLT2 inhibitors in children is still very limited, with only small case series and early exploratory studies available, and their use in pediatric nephrology remains uncommon. Importantly, no real-world data have been reported to date regarding the use of dapagliflozin specifically in pediatric IgAN. Our experience therefore provides novel clinical insight into this emerging therapeutic approach and underscores the need for dedicated pediatric trials to define long-term safety, renal outcomes and appropriate patient selection.

## ETHICAL DISCLOSURE

The study was conducted in accordance with the Declaration of Helsinki. As dapagliflozin was used off-label, written informed consent was obtained from parents or legal guardians prior to treatment initiation.

## Supplementary Material

sfaf395_Supplemental_File

## CONFICT OF INTEREST STATEMENT

None declared.
