# Supplementary material for: Dapaglifozin use in pediatric IgA nephropathy: a single-center real-life experience
Source: Clin Kidney J. 2025 Dec 13;19(1):sfaf395. doi: 10.1093/ckj/sfaf395 (PMC12789866; doi:10.1093/ckj/sfaf395)
Supplement: sfaf395_Supplemental_File [file sfaf395_supplemental_file.docx]

**Supplementary Tables**

**Supplementary Table 1. Baseline clinical, demographic, and histological characteristics of the study population**

| **Characteristic** | **Value** |
| --- | --- |
| **Number of patients** | 9 |
| **Age at dapagliflozin initiation, years** | 14 (13–15) |
| **Age at IgAN diagnosis, years** | 11 (10–13) |
| **Sex** | 7 males (77.8%),  2 females (22.2%) |
| **ACEi/ARB therapy** |  |
| – Ramipril 5 mg | 5 (55.6%) |
| – Ramipril 2.5 mg | 1 (11.1%) |
| – Ramipril 10 mg | 1 (11.1%) |
| – Telmisartan 10 mg | 2 (22.2%) |
| **Histology (MEST-C)** |  |
| – M1E0S0T0C0 | 8 (88.9%) |
| – M1E0S0T0C2 | 1 (11.1%) |
| **Immunosuppressive therapy** | 3 (33.3%) |
| **Dapagliflozin dose, mg/day** | 5 mg for all patients |

**Legend:** Values are presented as absolute numbers and percentages unless otherwise indicated. Age is expressed as median and interquartile range (IQR). MEST-C scores refer to the Oxford Classification of IgA nephropathy. All patients were treated with dapagliflozin 5 mg/day in addition to ACEi/ARB therapy.
**Abbreviations:** IgAN, IgA nephropathy; ACEi, angiotensin-converting enzyme inhibitor; ARB, angiotensin II receptor blocker.

**Supplementary Table 2. Hemodynamic, metabolic, and clinical outcomes during 6 months of dapagliflozin treatment**

| **Variable** | **T0** | **T1** | **T2** | **T3** |
| --- | --- | --- | --- | --- |
| **Systolic BP (mmHg)** | 116 (110–119) | 116 (112–126) | 122 (113–125) | 115 (111–120) |
| **Diastolic BP (mmHg)** | 60 (58–67) | 60 (56–63) | 64 (57–72) | 60 (59–68) |
| **Blood glucose (mg/dL)** | 87 (82–94) | 87 (81–92) | 89 (85–91) | 88 (81–95) |
| **BMI (kg/m²)** | 19.8 (18.7–28.6) | 20.6 (19.1–29.7) | 20.6 (19.9–29.7) | 20.8 (19.7–29.7) |
| **Urine output (mL/day)** | 1300 (1200–1700) | 1500 (1400–2000) | 1900 (1750–2700) | 2000 (1800–2550) |
| **Glucosuria, n (%)** | 0 (0%) | 8 (88.9%) | 8 (88.9%) | 7 (77.8%) |
| **Ketonuria, n (%)** | 0 (0%) | 1 (11.1%) | 1 (11.1%) | 2 (22.2%) |
| **Upper respiratory infections (IVAS), n (%)** | 0 (0%) | 4 (44.4%) | 4 (44.4%) | 5 (55.6%) |
| **Macroscopic hematuria, n (%)** | 2 (22.2%) | 0 (0%) | 0 (0%) | 0 (0%) |

**Legend:** Values are expressed as median and interquartile range (IQR) unless otherwise specified. Timepoints: T0 = baseline; T1 = 1 month; T2 = 3 months; T3 = 6 months.
**Abbreviations:** BP, blood pressure; BMI, body mass index.
